# Relationship of dose to vascular target volumes and local failure in pancreatic cancer patients undergoing neoadjuvant chemoradiation

**DOI:** 10.3389/fonc.2022.906484

**Published:** 2022-08-31

**Authors:** Bailey Nelson, Michelle Barrord, Kyle Wang, Nolan A. Wages, Mickaela Sudhoff, Jordan Kharofa

**Affiliations:** ^1^ Department of Radiation Oncology, University of Cincinnati, Cincinnati, OH, United States; ^2^ Department of Biostatistics, Virginia Commonwealth University, Richmond, VA, United States; ^3^ Massey Cancer Center, Virginia Commonwealth University, Richmond, VA, United States

**Keywords:** pancreas—adenocarcinoma, stereotactic body radiation therapy (SBRT), vasculature, radiation, neoadjuvant

## Abstract

**Objective:**

The objectives of this study were to evaluate whether dose to the vasculature is associated with local control after surgery in patients with borderline resectable (BLR) and resectable pancreatic cancer (PCA) receiving neoadjuvant radiation therapy (RT) and to identify a dose threshold for clinical use.

**Methods:**

Patients with BLR and resectable PCA treated with neoadjuvant RT were retrospectively reviewed. During this period, the institutional paradigm shifted from standard fractionation to hypofractionation/stereotactic body radiation therapy (SBRT). A vasculature clinical target volume (Vasc CTV) was contoured for each patient and defined as a 5-mm margin around the superior mesenteric artery (SMA) from its origin to the pancreatic head, the celiac artery from its origin to the level of the trifurcation and any involved vein. The Vasc CTV D95 was normalized to a 2-Gy equivalent dose to determine the optimal dose associated with optimal local failure-free survival (LFFS).

**Results:**

Forty-seven patients were included in the analysis. A Vasc CTV D95 of 32.7 Gy was the optimal cutoff for LFFS. Patients with Vasc CTV D95 Equivalent dose in 2 Gy per fraction (EQD2) >32.7 Gy had significantly longer LFFS compared to patients with Vasc CTV D95 EQD2 ≤32.7 Gy at 12 months (91% vs. 51%, respectively) and 24 months (86% vs. 12%, respectively). The median disease-free survival (DFS) for patients with EQD2 >32.7 Gy was 30.4 months compared to 14.0 months in patients with EQD2 ≤32.7 Gy (p = 0.01). There was no significant difference in overall survival (OS) between the two groups.

**Conclusions:**

During neoadjuvant treatment, dose to the Vasc CTV is associated with durability of local control (LC) after resection and should be intentionally included in the treatment volume with an EQD2 goal of 31–33 Gy.

## Introduction

Pancreatic cancer is a highly aggressive malignancy with high rates of mortality (1, 2). However, outcomes may be improved with multimodality therapy that includes a combination of surgery, chemotherapy, and radiation therapy. Neoadjuvant radiation therapy may be utilized for patients with resectable and borderline resectable disease to facilitate earlier initiation of systemic therapy, to improve the rate of margin-negative resection (R0), and to select patients most likely to benefit from surgical resection (3–9). Preoperative chemoradiation has also been shown to improve locoregional control, disease-free survival (DFS), and overall survival (OS) compared to surgery alone (3, 10).

Disease abutting the vasculature can limit the degree of resection, which can put these patients at high risk for harboring microscopic residual disease. Perineural invasion (PNI) is prominent in pancreatic cancer. Autonomic nerves course along the superior mesenteric and celiac arteries and are at high risk for microscopic spread, which can cause local failures along the vasculature following surgical resection (11–13). A literature review has shown that the incidence of PNI in pancreatic cancer can be as high as 100% (12). Yet, these vasculature regions at risk of microscopic disease are variably included within radiation treatment volumes on clinical trials (11, 14, 15). Radiation prescription dose in neoadjuvant treatment of borderline resectable and resectable pancreatic cancer can also vary. Commonly used neoadjuvant radiation regimens include standard fractionation to a dose of 50–50.4 Gy (5, 8), hypofractionation to a dose of 30–36 Gy (3, 6, 9), and stereotactic body radiation therapy (SBRT) to a dose of 30–40 Gy with or without an elective volume (15–17).

The optimal dose to the high-risk vascular volumes required to provide durable local control remains poorly understood. The aims of this study are to evaluate whether the dose to the vasculature is associated with local control after surgery in patients receiving neoadjuvant radiation and to identify a preferred dose threshold for clinical use.

## Methods

All consecutive patients diagnosed with borderline resectable or resectable pancreatic cancer of the pancreatic head, neck, or body treated with neoadjuvant radiation therapy from February 2011 to August 2020 at a single institution were retrospectively reviewed. Borderline resectable disease was defined as tumor contact with the superior mesenteric artery (SMA) or celiac artery (CA) less than or equal to 180°, short segment occlusion of the common hepatic artery (CHA), or tumor contact with the inferior vena cava (IVC) and/or involvement of the superior mesenteric vein (SMV) and portal vein (PV) that were considered reconstructable (18). Prior to initiation of radiation therapy, patients received 3 months of gemcitabine/nab-paclitaxel or FOLFIRINOX (bolus 5-fluorouracil, leucovorin, irinotecan, oxaliplatin) chemotherapy. Patients then underwent neoadjuvant radiation therapy with chemotherapy. Following completion of neoadjuvant therapy, patients underwent restaging imaging with computed tomography (CT) scans of their chest, abdomen, and pelvis. If imaging showed no evidence of metastases and resectable disease, patients proceeded with surgical resection. Patients did not receive adjuvant chemotherapy until diagnosed with progression.

**Figure 1:**
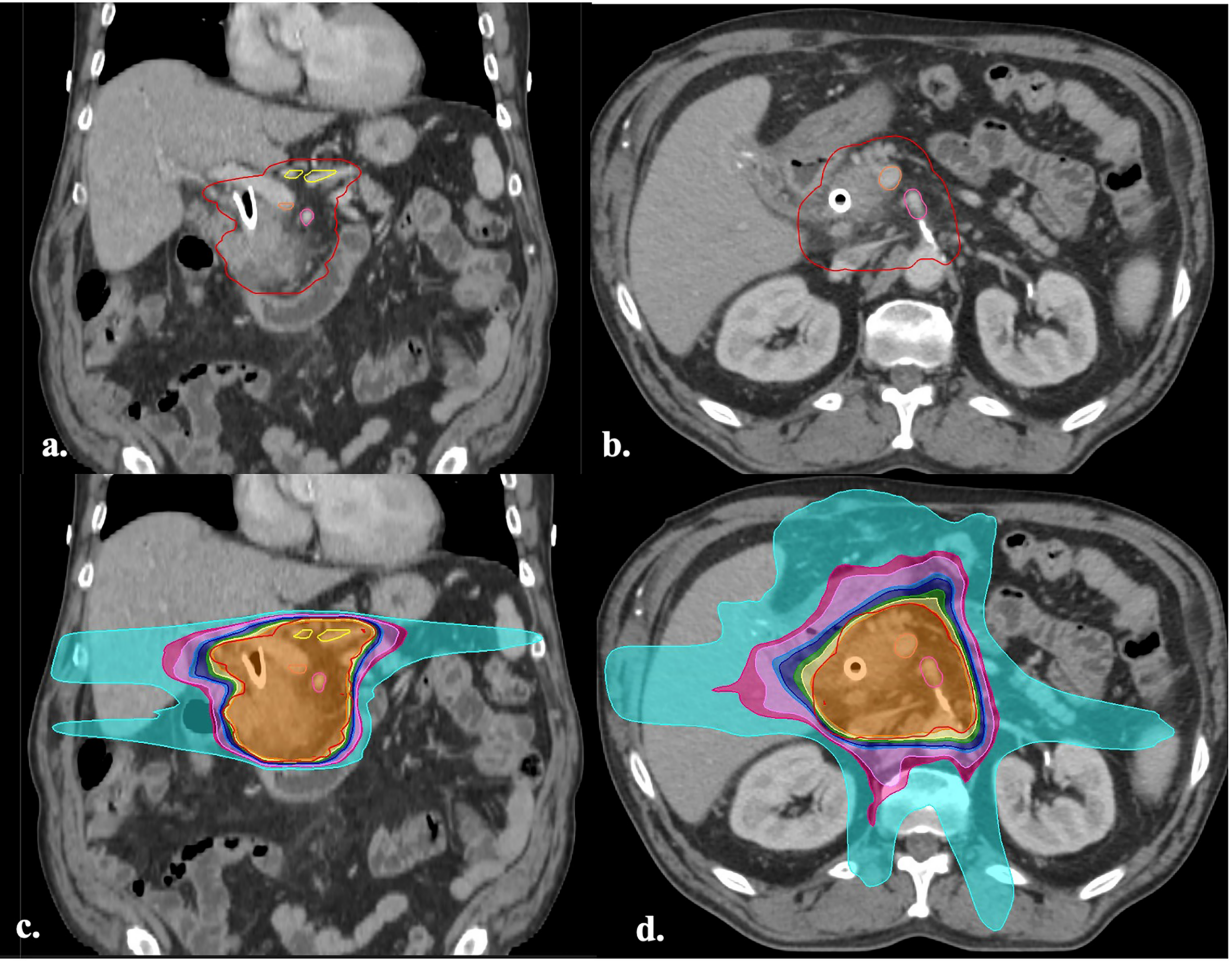
**(A, B)** Axial and coronal images of a CT simulation with the hypofractionation target volumes (36 Gy in 15 fractions) defined by previous works (8). The GTV (green), CTV (inner red), PTV (outer red), CA (yellow), SMV (orange), SMA (pink), can be visualized. **(C, D)** Axial and coronal images of the treatment plan for the same patient. The isodose lines are displayed in color wash and represent the following doses: 1,440 cGy (cyan), 2,160 cGy (hot pink), 2,314 cGy (light pink), 2,736 cGy (light blue), 2,880 cGy (dark blue), 3,240 cGy (green), 3,420 cGy (yellow), 3,600 cGy (orange), 3,780 cGy (red). GTV, gross target volume; CTV, clinical target volume; PTV, planning target volume; CA, celiac axis; SMV, superior mesenteric vein; SMA, superior mesenteric artery; Vasc CTV, vascular clinical target volume.

During the study period, the institutional preoperative radiotherapy approach underwent a paradigm shift from standard fractionation (50.4 Gy at 1.8 Gy/fraction) to hypofractionation (36 Gy at 2.4 Gy/fraction or 30 Gy at 3 Gy/fraction) both with concurrent chemotherapy. Some patients were treated with neoadjuvant SBRT on a single-institution phase 2 clinical trial (17). In the trial, the primary tumor planning target volume (PTV) alone was treated up to 33 Gy in 6.6 Gy per fraction. Following an interim analysis revealing several marginal failures, the protocol was amended to allow for an elective vasculature clinical target volume (Vasc CTV) that was treated up to 25 Gy in 5-Gy fractions (17). For patients treated with SBRT, the gross tumor volume (GTV) was defined as the primary tumor and entire circumference of the abutting vessel. An internal target volume (ITV) was added to account for respiratory motion of the tumor. The ITV was symmetrically expanded by 3 mm to create the primary PTV, which was treated up to 33 Gy in 6.6-Gy fractions. An optional elective Vasc CTV covered the entire pancreatic head and body, immediately abutting vessel, and the SMA and CA origin. This CTV was also expanded by 3 mm to create the elective PTV, which was treated up to 25 Gy in 5-Gy fractions. The target volumes for patients treated with conventionally fractionated and hypofractionated radiation were consistent with previous works (8). The primary CTV broadly included the primary tumor ITV accounting for respiratory motion, SMA (from aortic origin inferiorly to the pancreatic head), celiac axis, and suspicious lymph nodes. The CTV was expanded by 5–10 mm to create a PTV. The porta hepatis was not routinely covered. An example of hypofractionation target volumes and treatment plan are shown in **Figure 1**. The evolution of dose/fractionation schemes used during the study period and variation in target volumes provides an opportunity to examine local control as a function of standardized dose to the high-risk vascular regions.

**Figure 2:**
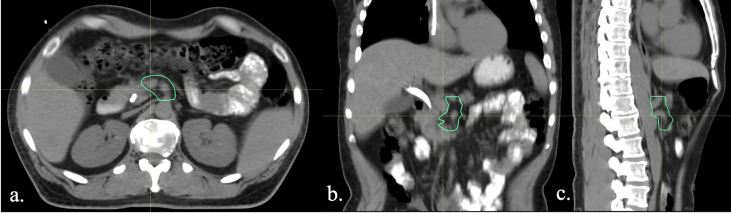
**(A)** Axial view, **(B)** coronal view, and **(C)** sagittal view of the Vasc CTV.

A customized high-risk vascular target volume, termed “Vasc CTV,” was retrospectively contoured on the planning CTs of all patients treated with neoadjuvant radiation therapy and surgical resection during the study period. The area at highest risk of local recurrence was determined to be along the mesenteric vasculature based on previous works evaluating patterns of failure data (11). The Vasc CTV was delineated as a 5-mm margin around the SMA from its origin to the pancreatic head, the CA from its origin to the level of the trifurcation and any involved vein (**Figure 2**). The radiation dose to 95% of this volume (D95) was calculated on treatment plans. In order to account for differences in total radiation dose and fraction size, a 2-Gy dose per fraction equivalent was calculated using the EQD2 method for all patients using an alpha/beta ratio of 10 for tumor.

### Statistical analysis

The primary endpoint was the duration of local failure-free survival (LFFS) as defined by time from surgery to identification of local failure on imaging. Local failure was defined as recurrence of disease postoperatively within standard conventional treatment volumes (8) on CT imaging follow-up. CT imaging was registered and aligned to remnant postoperative vasculature including the aorta, CA, and SMA. Local recurrences were contoured and evaluated on the initial treatment plan. Patients who were not diagnosed with local failure on their most recent CT imaging were censored. The Kaplan–Meier product limit estimator was used to estimate survival distributions for LFFS, DFS, and OS. OS was defined as time from surgery to date of death or last follow-up visit. DFS was defined as time from date of diagnosis to date of disease progression. Normalized dose to the Vasc CTV was dichotomized into “high” and “low” classifications using the method of Contal and O’Quigley (19) to determine the optimal cutoff for a continuous covariate with respect to a time-to-event outcome (LFFS). Multivariate Cox proportional hazards modeling was used to evaluate the association of normalized dose to the Vasc CTV with local recurrence-free survival, adjusting for arterial abutment. Cox proportional hazards assumptions were tested using Schoenfield residual analysis using a level of significance of 0.01. Associations were considered significant for two-sided p-values ≤0.05. Statistical analysis was performed using R software version 4.0.2.

## Results

A total of 75 patients were treated with neoadjuvant radiation therapy and retrospectively reviewed. Forty-seven patients had surgical resection and were included in the local failure analysis. Patient characteristics can be seen in **Table 1**. Twenty-six of the patients (55%) were considered to have borderline resectable disease at diagnosis, and the remaining 21 (45%) patients were considered to have resectable disease. Twenty patients (43%) were treated with standard fractionation, 15 patients (32%) were treated with hypofractionation, and 12 patients (25%) were treated with SBRT. Thirty-one patients (66%) were treated with gemcitabine/abraxane, and 15 (32%) were treated with FOLFIRINOX. The median follow-up from the time of surgery was 29 months (range: 10–120 months). Twelve (26%) patients had a local failure event. Two (4%) patients had synchronous local and distant recurrence. The median Vasc CTV D95 was 38.2 Gy (range: 0.34–50.2 Gy).

**Table 1:** Patient characteristics.

**Age, years (**mean, range)	63 (37–86)
**Sex** (n, %)	
Women Men	23 (49) 24 (51)
**Resectability** (n, %)	
Borderline Resectable Resectable	26 (55) 21 (45)
**CA 19-9 U/ml** (mean, range)	888 (0.8 – 9,300)
**Chemotherapy** (n, %)	
Gemcitabine/Abraxane FOLFIRINOX Gemcitabine/Cisplatin	31 (66) 15 (32) 1 (2)
**RT Fractionation** (n, %)	
50.4 Gy/28 fractions 50 Gy/25 fractions 36 Gy/15 fractions 36 Gy/12 fractions 30 Gy/10 fractions 33 Gy/5 fractions 33 Gy/25 Gy/5 fractions	14 (29.8) 6 (12.8) 5 (10.6) 2 (4.3) 8 (17.0) 6 (12.8) 6 (12.8)

RT, radiation therapy.

Five patients had perivascular failures. Two patients treated with 33 Gy in five fractions had a recurrence at the CHA. One of the aforementioned patients had an elective volume treated up to 25 Gy. Another patient treated with 33/25 Gy in five fractions had a local failure at the gastroduodenal artery. One patient treated with 50.4 Gy in 28 fractions had local failure at the gastroduodenal artery, PV, and SMV. The fifth patient had a recurrence at the celiac artery, porta hepatis, and retroperitoneal vasculature after 33 Gy in five fractions.

Using the Contal and O’Quigley (19) method, a Vasc CTV D95 of 32.7 Gy was the optimal cutoff for LFFS. Twelve (26%) of the patients were considered to have a Vasc CTV D95 EQD2 ≤32.7 Gy, and 35 (74%) of the patients were considered to have a Vasc CTV D95 EQD2 >32.7 Gy. The baseline characteristics between patients receiving high and low dose to Vasc CTV were compared. There were no significant differences in arterial abutment, baseline cancer antigen 19-9 (CA 19-9) levels, sex, age at time of diagnosis, nodal positivity, or R1 resection rate (**Table 2**).

**Table 2:** Patient and tumor characteristics.

	Patients with Vasc CTV D95 ≤32.7 Gy EQD2 (n = 12)	Patients with Vasc CTV D95 >32.7 Gy EQD2 (n = 35)	p-value
Arterial abutment (n, %)	4 (33.3)	12 (34.3)	1.000
LN positive (n, %)	3 (25.0)	13 (38.2)	0.635
R1 resection (n, %)	2 (18.2)	4 (11.4)	0.947
CA 19-9 U/ml (mean, SD)	497 (600)	550 (1,263)	0.894
Age at diagnosis, years (mean, SD)	63 (13)	63 (8)	0.901
Women (n, %)	8 (66.7)	15 (42.9)	0.276
Chemotherapy (n, %)			
FOLFIRINOX Gemcitabine/Abraxane Gemcitabine/Cisplatin	6 (50) 6 (50) 0 (0)	9 (26) 25 (71) 1 (3)	0.250

SD, standard deviation; LN, lymph node. FOLFIRINOX is a common chemotherapy and the different drugs do not need to be listed.

Patients with Vasc CTV D95 EQD2 >32.7 Gy had significantly longer LFFS compared to patients with Vasc CTV D95 EQD2 ≤32.7 Gy at 12 months (91% vs. 51%, respectively) and 24 months (86% vs. 12%, respectively). The median LFFS for patients with Vasc CTV D95 EQD2 >32.7 Gy was not reached compared to 12.9 months (95% CI 5.5–not reached) in patients with a Vasc CTV D95 EQD2 ≤32.7 Gy (log-rank p < 0.0001). After adjusting for arterial abutment, patients with a Vasc CTV D95 EQD2 >32.7 Gy showed significantly longer LFFS [hazard ratio (HR) = 0.10, p = 0.001].

DFS was significantly increased in patients with a Vasc CTV D95 >32.7 Gy. The median DFS for patients with EQD2 >32.7 Gy was 30.4 months compared to 14.0 months in patients with EQD2 ≤32.7 Gy (log-rank p = 0.01). There was no significant difference in OS between the two groups. The median OS for patients with a Vasc CTV D95 EQD2 >32.7 Gy was 39.8 months (95% CI 32.6–N/A) compared to 32.2 months (95% CI 19.9–N/A) in patients with Vasc CTV D95 EQD2 ≤32.7 Gy (log-rank p = 0.4).

## Discussion

Neoadjuvant radiation therapy is utilized in borderline resectable and resectable pancreatic cancer to improve the R0 resection rate, provide durable local control, and improve OS (10). However, the optimal radiation dose and radiation target volume remain undefined, and target volume delineation is not standardized on clinical trial protocols (10, 14). Bluemel et al. (14) compared the coverage of high-risk vascular regions using contouring guidelines recommended in three contemporary trials in borderline resectable pancreatic cancer, Alliance A021101, Alliance 021501, and PREOPANC, using DICE analysis (11, 14). In these trials, Boolean methods of expansion from the GTV were used to generate CTV target volumes (GTV + 1–3 cm) rather than intentional coverage of the vascular regions at risk. The analysis showed high variability in DICE coefficients (range 0.11–0.99), indicating significant variability in coverage of these high-risk regions with these methods of target construction (14). The American Society for Radiation Oncology (ASTRO) formed a task force to address questions focused on radiation therapy for pancreatic cancer (20). The task force conditionally recommended conventionally fractionated and SBRT for neoadjuvant treatment of borderline resectable pancreatic cancer. The guidelines list a range of suggested primary tumor radiation doses but does not provide specific guidance on nodal and vasculature coverage or dose for neoadjuvant SBRT (20).

Previous data have shown high rates of local recurrence at the vasculature margin to the primary tumor PTV (15, 17); therefore, recommendations on vasculature target delineation and radiation dose are needed. There is also an emerging trend toward hypofractionated regimens in the preoperative setting. The PREOPANC trial randomized patients with resectable or borderline resectable pancreatic cancer to receive either preoperative chemoradiation (36 Gy in 15 fractions) followed by surgery and adjuvant chemotherapy or immediate surgery followed by adjuvant chemotherapy (3). R0 resection rate, DFS, and locoregional failure-free interval improved with preoperative chemoradiation (3). Preoperative treatment was also associated with lower rates of PNI, venous invasion, and pathologic lymph nodes (3). A recent update analysis of the trial shows that both 3-year OS and 5-year OS are statistically significantly improved with preoperative chemoradiation vs. immediate surgery (10). In addition, there is increased interest in ablative radiotherapy regimens for patients with unresectable disease (21, 22). There remains controversy and debate regarding dose and target delineation to the high-risk vascular space in these settings.

Given the significant heterogeneity in both target delineation of the high-risk vascular targets and the various fractionation schemes in use (**Table 3**), the goal of this study was to determine whether a standardized dose equivalent to this region in the preoperative setting was associated with improvement in the durability in local control following resection. Our results indicate that treatment of a vascular CTV to an EQD2 of at least 32.7 Gy is associated with improvement in LFFS and DFS. The data suggest that vasculature coverage and dose to the vasculature are both important for adequate disease control. Commonly used treatment regimens that achieve an EQD2 of at least 32.7 Gy (assuming α/β = 10) are 50.4 Gy in 1.8-Gy fractions, 50 Gy in 2-Gy fractions, 45 Gy in 1.8-Gy fractions, 36 Gy in 2.4-Gy fractions, and 33 Gy in 6.6-Gy fractions.

**Table 3:** Elective target coverage in select neoadjuvant trials.

Trial	Elective Target Volume	Elective Target Dose	Elective Target EQD2 (α/β = 10)
Preoperative gemcitabine-based chemoradiation for patients with resectable adenocarcinoma of the pancreatic head^27^	Pancreaticoduodenal, porta hepatis, superior mesenteric, and celiac axis lymph nodes with 2-cm block margin	30 Gy in 10 fractions	32.5 Gy
PREOPANC^3^	GTV = primary tumor + pathologic lymph nodes; CTV = GTV + 5 mm uniform expansion	36 Gy in 15 fractions	37.2 Gy
Alliance 021101^5^	GTV = primary tumor + regional adenopathy >1 cm in size; CTV = GTV + 1 cm	50.4 Gy in 28 fractions	49.56 Gy
Alliance 021501^30^	TVI was created for each vessel in contact with the tumor including the PV, SMV, SMA, CA, and CHA	33 Gy in 5 fractions, or 25 Gy in 5 fractions	45.65 Gy 31.25 Gy
Total Neoadjuvant Therapy with FOLFIRINOX Followed by Individualized Chemoradiotherapy for Borderline Resectable Pancreatic Adenocarcinoma^23^	CA, porta hepatis, SMA, SMV, and para-aortic groups	25 GyE in 5 fractions with protons 30 Gy in 10 fractions with photons 50.4 Gy in 28 fractions	31.25 Gy 32.5 Gy 49.56 Gy

TVI, tumor vessel interface.

Twelve patients in our series received 25 Gy in five fractions to the elective PTV. This regimen has an EQD2 of 31.25 Gy, which falls near the D95 threshold of 32.7 Gy. In a phase 2 trial of patients with borderline resectable pancreatic cancer, patients with resolution of vascular involvement after eight cycles of FOLFIRINOX received 5 GyE × 5 fractions with protons and patients with persistent vascular involvement received long-course chemoradiation (23). The CTV included elective nodal coverage including the celiac, porta hepatis, SMA, SMV, and para-aortic regions (24). Median progression-free survival (PFS) was 48.6 months in resected patients, and negative surgical margins were achieved in 86% of patients (23). The 25 Gy in 5-Gy fraction regimen was shown to achieve favorable local control in patients with resolution of vascular involvement after induction chemotherapy. In addition, for patients with persistent vascular abutment, a simultaneous boost technique was used to the vascular/tumor interface of 58.8 Gy in 28 fractions. Also, intraoperative radiation was used at the discretion of the surgeon. These factors may have improved outcomes by giving increased doses to at-risk vasculature (23). In addition, a recent propensity matched analysis of patients treated with SBRT with or without an elective CTV (25 Gy/5 fractions) revealed a decrease in the 24-month cumulative incidence of local progression with elective target coverage (22.6% vs. 44.6%, p = 0.021) (25). Therefore, 25 Gy to the elective vascular region may be an appropriate treatment option for well-selected patients when 5-fraction regimens are used in the neoadjuvant setting.

It is unlikely that a randomized trial will evaluate elective dose schemes in pancreatic cancer. It is our opinion that intentional target delineation of this space is reasonable in the preoperative setting. Our findings indicate that the dose to this space is related to the durability of local control. This is consistent with prior patterns of failure analyses (11, 15, 17). Boolean methods of target generation of tumor + margin may not appropriately cover this high-risk region. Future trials incorporating radiation in the neoadjuvant setting should ideally include guidance for clinicians on elective target delineation and robust quality assurance mechanisms to ensure accurate delivery of treatment similar to what has been used for adjuvant radiotherapy in pancreatic cancer trials (26). A variety of dose/fractionation schemes may be reasonable to provide adequate local control to the vascular regions at risk. Based on the analyses herein and prior published works (3, 6, 17, 21, 27), an EQD2 dose of approximately 31–33 Gy would be a reasonable approach.

This study is limited in that it was performed at a single institution, the data were collected retrospectively, and only 47 resected patients were included in the local failure analysis. Given the sample size, we are unable to account for potential interactions with chemotherapy regimens over the study period. However, all but one patient received FOLFIRINOX or gemcitabine/abraxane as neoadjuvant treatment and there does not appear to be major differences in survival outcomes in these regimens in the neoadjuvant setting (28). The study would be strengthened by a separate validation cohort to evaluate whether the EQD2 cutoff of 32.7 Gy to the vascular space decreases local recurrence rate. With additional cohorts, the optimal cutoff may vary slightly from this metric.

## Data availability statement

The original contributions presented in the study are included in the article/supplementary material. Further inquiries can be directed to the corresponding author.

## Ethics statement

The studies involving human participants were reviewed and approved by University of Cincinnati IRB. Written informed consent for participation was not required for this study in accordance with the national legislation and the institutional requirements.

## Author contributions

BN and MB equally contributed to this work and share first authorship. All authors contributed to the article and approved the submitted version.

## Conflict of interest

The authors declare that the research was conducted in the absence of any commercial or financial relationships that could be construed as a potential conflict of interest.

## Publisher’s note

All claims expressed in this article are solely those of the authors and do not necessarily represent those of their affiliated organizations, or those of the publisher, the editors and the reviewers. Any product that may be evaluated in this article, or claim that may be made by its manufacturer, is not guaranteed or endorsed by the publisher.
